# Temporary Abdominal Closure Combined With an Irrigating System Utilizing Hypochlorous Acid Solution to Decrease Abdominal Mucopurulence

**Published:** 2018-02-26

**Authors:** Marc R. Matthews, Asia N. Quan, Alexandra S. Weir, Kevin N. Foster, Daniel M. Caruso

**Affiliations:** ^a^Department of Surgery, Maricopa Integrated Health System, Phoenix, AZ; ^b^Burn/Trauma Critical Care Clinical Pharmacist, Department of Pharmacy, Maricopa Integrated Health System, Phoenix, AZ; ^c^Department of Surgery, Arizona Burn Center, Maricopa Integrated Health System, Phoenix, AZ

**Keywords:** negative pressure wound therapy, temporary abdominal closure, VAC veraflo, abthera device, vashe solution

## Abstract

**Introduction:** Leaving the abdominal cavity open is a well-described and frequently utilized technique in the treatment of severe intra-abdominal sepsis. Irrigation through a negative pressure wound therapy device is a technique employed to assist in the closure of wounds as well as the reduction of bacterial contamination. Furthermore, hypochlorous acid has been found to be safe and effective in microorganismal elimination from extremity wounds. There is no literature regarding the infusion of hypochlorous solution into the abdominal cavity for intra-abdominal sepsis or mucopurulent abscesses or biofilm. **Objectives**: A 47-year-old man with granulomatosis polyangiitis was started on weekly rituximab. After 4 infusions, skin sloughing, ultimately diagnosed as toxic epidermal necrolysis, developed. During the hospital course, he developed sepsis and bowel perforation necessitating an exploratory laparotomy. The abdomen was left open with a temporary abdominal closure using the Abthera open abdomen negative wound therapy device; however, the abdomen remained infected with visually diffuse, thickening mucopurulence despite multiple washouts. Therefore, a VAC Vera-Flo irrigation device was combined with the Abthera open abdomen negative wound therapy device and cyclical irrigation of hypochlorous acid. After 72 hours, the purulence visually was improved and no adverse events were recorded with the placement of intra-abdominal hypochlorous acid. **Conclusions**: The combination of two medical devices for the intra-abdominal instillation of irrigation is considered “off-label use” from the manufacturer's recommendations. In addition, the repeated instillation of hypochlorous acid solution has not been described but was noted to have visually decreased the contaminated effluent within the intra-abdominal fluid.

Leaving the abdominal cavity open is a well-described and frequently utilized technique to treat severe intra-abdominal sepsis and for damage control laparotomy.^1^ The ultimate objective of the temporary abdominal closure (TAC) is relief of intra-abdominal hypertension, which improves visceral blood flow, decreases edema, reduces open abdominal size, and removes intra-abdominal fluid, including mucopurulent exudate and biofilm.^2^^,^^3^ While multiple TAC system devices have been described, the Abthera Open Abdomen Negative Pressure Wound Therapy (ABNPWT) System (Acelity, San Antonio, Tex) is an effective TAC method.^4^^,^^5^ Irrigation through a negative pressure wound therapy (NPWTi) device is a technique recently employed to assist in the closure of wounds.^6^^,^^7^ Irrigation through the VAC Veraflo system (Acelity) added to the ABNPWT is a further adjunct wherein a wound, including the open abdomen, may be irrigated using a timed, pre-programmed lavage of a defined quantity without having to reopen the ABNPWT, sparing recurrent trips to the operating room (OR). This also minimizes pain and the effects of repeated general anesthetics-like medications while performing frequent ABNPWT replacement at the bedside.

Hypochlorous acid (HOCl, Vashe, SteadMed, Fort Worth, Tex) has been found to be safe and effective in microorganismal elimination from wounds.^8^ The majority of such wound beds are on extremities or on the torso at the skin, subcutaneous tissue, or fascia/muscle level; there is no literature regarding the infusion of HOCl solution into the abdominal cavity for intra-abdominal sepsis or mucopurulent abscesses or biofilm. Herein is described an immunocompromised patient with an open abdomen and intra-abdominal fungal sepsis. Despite aggressive surgical care and intravenous antimicrobials, the patient could not clear his intra-abdominal mucopurulent drainage that only seemed to worsen over time, irrespective of his frequent abdominal washouts. He was subsequently treated with the novel combination of two technologies, VAC Veraflo and ABNPWT, for the repeated introduction of intra-abdominal HOCl solution.

## CASE REPORT

A 47-year-old man with granulomatosis polyangiitis (GPA) (formerly known as Wegener's granulomatosis) was admitted to a local medical center for new-onset hemoptysis secondary to diffuse alveolar hemorrhage (DAH) that eventually required a tracheostomy for respiratory failure. During that hospitalization, he was treated with extracorporeal membrane oxygenation (ECMO), plasmapheresis, intravenous methylprednisolone, intravenous immunoglobulin (IVIG), and rituximab (Genentech, San Francisco, Calif) weekly for 1 month. The patient developed rapidly progressing skin sloughing. Results of a skin biopsy were consistent with Steven-Johnson syndrome thought to be secondary to rituximab, which was immediately stopped.

**Figure 1:**
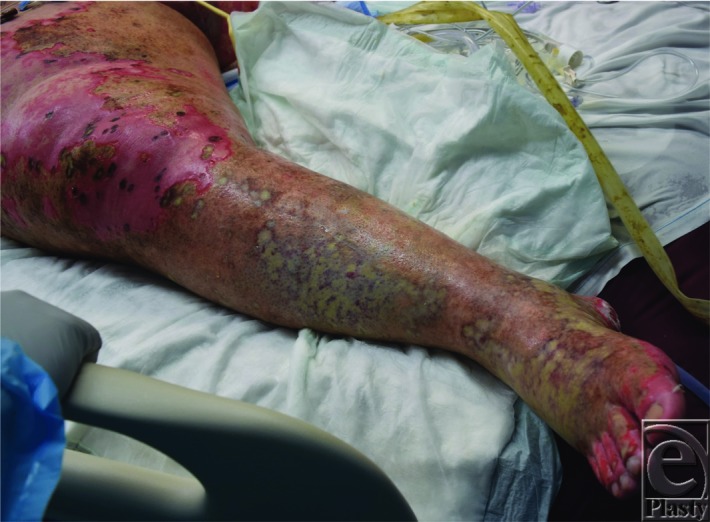
Initial necrotic wounds of the right lower extremity prior to the first operative debridement with evidence of full-thickness lesions caused by the granulomatosis polyangiitis (GPA) (formerly known as Wegener's granulomatosis).

The patient was promptly transferred to an American Burn Association–verified burn center intensive care unit (ICU) where his methylprednisolone was continued for the treatment of GPA and initiated on a second 3-day course of 1g/kg/24 hours IVIG. On arrival his wounds were reassessed to be 45% total body surface area consistent with TEN and were treated with xeroform and bacitracin dressings to his bilateral upper/lower extremities and torso. On hospital day (HOD) 5, methylprednisolone was decreased to 30 mg daily in an attempt to wean the steroids. The patient was taken to the OR for debridement and application of porcine xenograft to his open wounds. Central lines were cultured and then removed on HOD 8 and 9 and resulted with *Pseudomonas* spp, as well as *Enterococcus*. Peripheral blood cultures drawn on HOD 10 were negative. Bronchoalveolar lavage performed on HOD 10 demonstrated *Pseudomonas spp*. The patient developed multiple courses of sepsis starting on HOD 10 requiring treatment with intravenous antibiotics, vasopressors, and an increase in methylprednisolone back to 50 mg IV daily. All the wounds converted to full-thickness necrotic lesions for which he repeatedly underwent debridement (Figures 1 through 4). The patient eventually underwent bilateral lower extremity fascial debridements that were covered with negative pressure wound therapy using silver-impregnated sponges (KCI, a company of Acelity).

During one septic episode, the patient had increasing naso-gastric tube output and a computed axial tomographic scan of the abdomen and pelvis demonstrated a large amount of fluid and possible free air. An ultrasound-guided paracentesis was performed, removing several liters of succus entericus. At subsequent exploratory laparotomy, a perforated portion of small bowel that was resected, leaving the small bowel in temporary discontinuity. The patient's abdomen was left open with a TAC using the ABNPWT system. The patient returned to the OR multiple times for abdominal washout and TAC and intra-abdominal fluid culture obtained on HOD 22 resulted *Candida utilis* and *Pseudomonas aeruginosa*. Micafungin was empirically initiated, which, based on pathology, was escalated to liposomal amphotericin B pending fungal speciation. Bilateral thigh tissue cultures taken on HOD 27 resulted in *Candida utilis, Blastoschizomyces capitatus,* and *Aspergillus* species.

**Figure 5:**
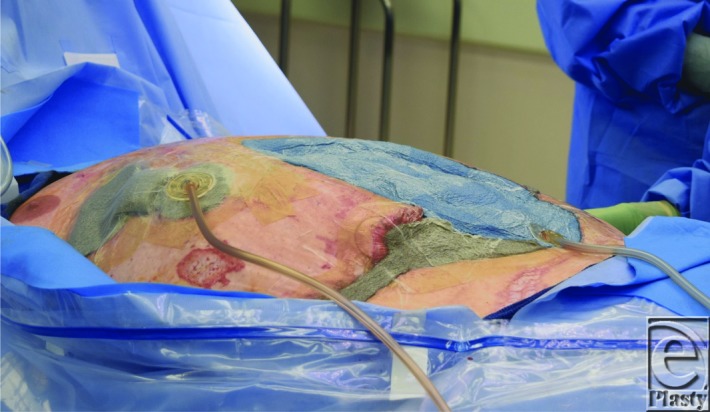
VAC Veraflo tracking device (inferior dual lumen) over the Abthera system (blue sponge) device placed for intra-abdominal instillation of Vashe solution. The VAC dressing (silver sponge) on the right upper and lower quadrant immediately adjacent under negative pressure wound therapy is a single lumen device.

**Figure 6:**
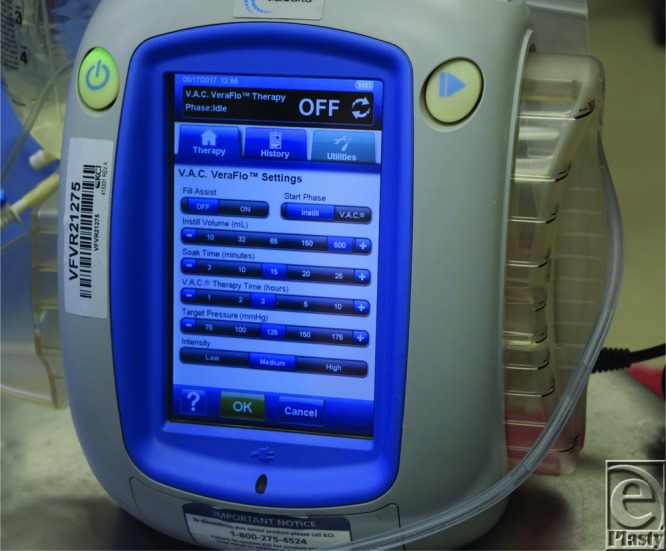
The VAC Veraflo suction device display showing the settings for instillation as well as negative pressure wound therapy. Fill Asist is turned “OFF” and the Start Phase reads “Instill”. The Instill Volume (mL) reads 500 for the amount to be instilled into the abdomen and the “Soak Time (minutes)” is set at “15 minutes.” VAC Therapy Time (hours) is reading set at “3.” The Target Pressure (mmHg) reads “125” and Intensity is read at “Medium.” The side canister is currently a 500 mL container but was increased to the larger capacity container of 1000 ml.

The patient returned to the OR every 2 to 3 days for TAC change during which a small bowel re-anastomosis was performed. During the third washout, the abdomen was noted to have a visually diffuse, heavy mucopurulent exudate, not emanating from the bowel anastomosis, despite frequent abdominal washouts. It was decided to combine a VAC Vera-Flo for irrigation (KCI, a company of Acelity) therapy with the ABNWPT (Figure 5). Initially, 500 mL NS was instilled for 30 minutes (Figure 6); all subsequent cycles utilized HOCl solution through the VAC Vera-Flo device into the abdomen, 500 mL over 30 minutes, and removed every 3 hours with the VAC Vera-Flo device. Forty-eight hours after the switch to the VAC Veraflo/HOCl irrigation, it was noted in the OR that there was visually no further purulent drainage collecting from the abdominal gutters or emanating from the ABNPWT intra-abdominal device. The bowel appeared normal, with no duskiness or necrosis noted. Additionally, the abdominal serosa appeared normal, without erythema or violaceous appearance. During the abdominal HOCL irrigations, no anaphylactic, tachycardic, or hypotensive events were noted.

After the last trip to the OR, the patient and his family opted to transition to comfort care and the patient expired.

## DISCUSSION

TEN approximates a partial thickness-like burn injury; however, wounds may continue to evolve into full-thickness-like burns requiring a full-thickness excision, and at worse an excision of the skin and subcutaneous tissue down to the level of the fascia. Early identification of TEN and withdrawal of the offending agent are essential. Unfortunately for patients with large surface area burns or burn-like lesions requiring fascial excision, there is a high mortality rate from up to 75% especially with additional organ failures.^9^ Furthermore, if the abdominal cavity is subsequently opened in any burn patient, the prognosis remains grim. Recent reports from the United States Army Institute of Surgical Research (USAISR) Burn Center have placed the mortality rate for burn patients requiring a TAC at 68%.^10^

ABNPWT using subatmospheric pressures has been shown to be effective in removal of mucopurulent exudate and infectious material,^11^^,^^12^ improving wound healing by reducing edema, improving granulation tissue production, increasing blood flow, and contraction of acute and chronic wounds prior to closure. Recently, NPWTi has been utilized in the open abdomen as a timed, pre-programmed delivery of an irrigation bolus with a specified wound dwell time prior to removal by negative pressure.^13^ The frequency of instillation, in-wound irrigation dwell time, and type and size of irrigation is adjustable. The NPWTi device can assist in removal of necrotic or mucopurulent debris secondary to infection or effluent from a perforated bowel. Foam dressings over a wound bed have led to improved outcomes; irrigation is believed to facilitate the removal of thick wound exudate and infectious material.^3^^,^^14^ In a large series of patients with severe abdominal sepsis with various levels of organ failure from infection, Sibaja and colleagues^15^ described intra-abdominal NPWTi in 48 patients using NS and found improved morbidity and mortality as compared to standard NPWT. D'Hondt and colleagues^16^ described the use of NPWTi to treat abdominal sepsis secondary to a laparostomy after traditional management had failed. Recently, the VAC Veraflo device has been introduced, with the premise that increased wound bed irrigation will help mitigate infection, allowing for earlier wound closure. Another case report describing patients with deep wound infections utilized infusion of antibiotics (colistin-rifampicin solution) through the VAC Veraflo device, and noted a reduction in bacterial load and exudate.^17^

HOCl acid has also been described as a wound cleanser. It has been shown to significantly reduce bioburden in peripheral open wounds, thereby assisting in their closure. However, intra-abdominal placement of HOCl has never been described to treat the open septic abdomen. The combination of the ABNPWT device system and the VAC Veraflo device allows for delivery of a set amount of irrigation in a timed manner. This combination in a patient with a septic abdomen proved pivotal in the resolution of the gross mucopurulence visualized in the abdomen.

Within this patient's hospital course, all therapeutic options had been thoroughly investigated and exhausted, including the use of TAC, potent intravenous antibiotics for sepsis and intravenous antifungals for his abdominal fungal infections. It is likely that the immunosuppression resulting from the necessary treatment of the patient's comorbidities was a contributing factor in the difficulties of clearing his infections. In the end, the only clinical options available to salvage the patient were off-label medical avenues. Application of the ABNPWT to remove intra-abdominal fluid was initiated, but repeated heavy growth of thick mucopurulent exudate found with visual inspection and on cultures was the impetus to escalate to more aggressive, off-label measures. The unique multimodal approach of combining the VAC Veraflo with the ABNPWT and intra-abdominal irrigation with NS may have been enough, however, the fact that HOCl decreases the bioburden of topical wounds was considered. Such a use of ABNWPT and the VAC Veraflo combination as well as for the HOCl irrigation into the abdominal cavity would be noted as off-label use. HOCl has been studied *in vitro* and found to have a rapid bactericidal and fungicidal effect against many pathogens, and has been safely used to enhance the treatment of necrotizing soft tissue wounds.^18^^,^^19^ A literature review did not reveal prior cited articles using intra-abdominal HOCl or any contraindications to this novel mode of administration. During the abdominal laparotomy following 72 hours of HOCl irrigation, upon visual inspection, the mucopurulence was quelled. No evidence of tachycardia, hypotension, hyperpyrexia/hypothermia, or anaphylactoid reactions was noted in the patient while receiving the intra-abdominal HOCl irrigation.

In the future, a case series with nonimmunologically challenged patients to assess for potential reactions in patients receiving intra-abdominal HOCl would be useful. Future clinical trials using variable amounts of irrigation up to 1 L to compare the differences in bacterial clearance between HOCl solution to NS would also be useful. When considering these endeavors, it is necessary to recognize that as long as the occlusive dressings seal remains intact to the epidermal layer, abdominal bladder pressures need to be monitored to avoid the development of an intra-abdominal compartment syndrome. In addition, the patients would need to be monitored for development of hypothermia, hyperpyrexia, or any anaphylactoid reaction including monitoring of other vital signs.

## CONCLUSIONS

We describe the combination of the ABNPWT and the VAC Veraflo devices plus the intra-abdominal use of HOCl solution to the open septic abdomen as two off-label interventions in a severely ill and immunocompromised patient. We recommend further studies to elucidate the use of these two technologies and the utility for intra-abdominal placement of HOCl solution to help in clearing the septic abdomen of opportunistic microorganisms.

## Figures and Tables

**Figure 2:**
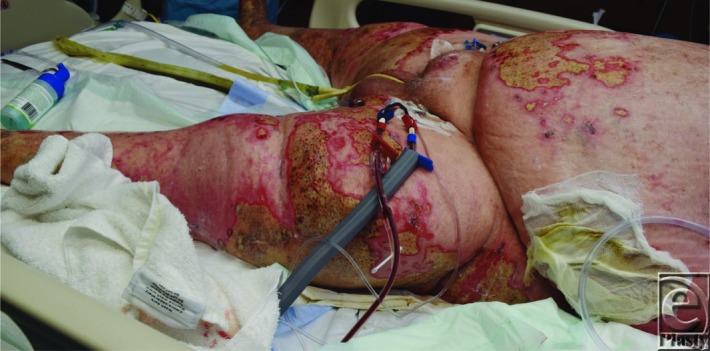
Initial necrotic wounds of the bilateral lower extremities and abdomen prior to the first operative debridement caused by the granulomatosis polyangiitis (GPA) (formerly known as Wegener's granulomatosis).

**Figure 3:**
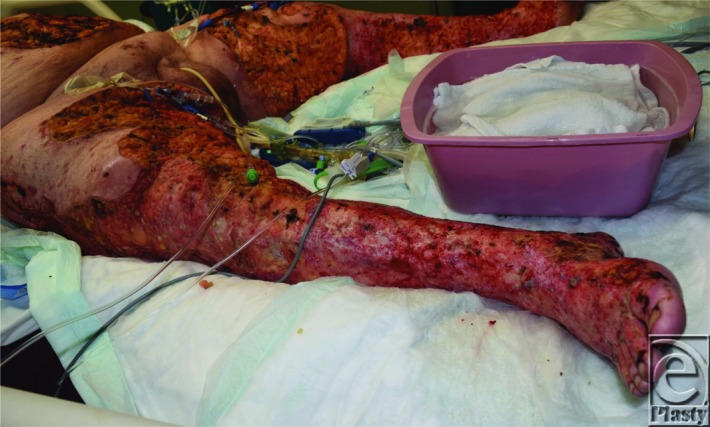
Open wounds to the bilateral lower extremities and abdomen after the first operative debridement for the necrotic full-thickness lesions.

**Figure 4:**
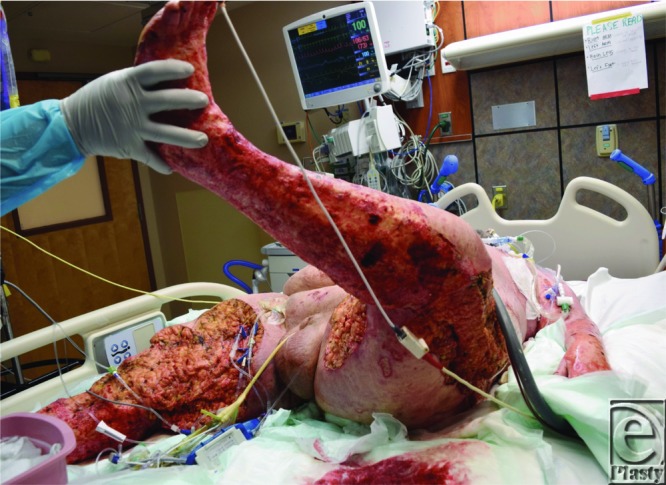
Open wounds to the bilateral lower extremities after the first operative debridement for the necrotic full-thickness lesions.
